# Unilateral Scrotal Darkening as an Unusual Neonatal Finding: A Case Report

**DOI:** 10.7759/cureus.117753

**Published:** 2026-10-11

**Authors:** Catarina B Azevedo, Lucinda Amorim-Delgado, Sara Nogueira Machado, Sofia Martinho, Sandra Rodrigues

**Affiliations:** 1 Department of Pediatrics, Unidade Local de Saude do Alto Ave, Guimarães, PRT; 2 Department of Pediatric Surgery, Unidade Local de Saúde de Braga, Braga, PRT; 3 Department of Pediatrics, Unidade Local de Saúde do Tâmega e Sousa, Penafiel, PRT

**Keywords:** laparoscopic orchiopexy, neonatal testicular torsion, newborn surgery, spermatic cord torsion, testicular diseases, testicular ultrasonography, unilateral orchiectomy

## Abstract

Neonatal testicular torsion (NTT) is a rare condition that carries a substantial risk of ischemia, necrosis, and subsequent testicular loss. When the event occurs before birth, it is classified as prenatal torsion, a typically non-acute entity with an incompletely understood pathophysiology and limited potential for testicular salvage. Clinically, prenatal torsion often presents as a painless, firm, and enlarged testis and may remain unrecognized without careful neonatal examination. Although color Doppler ultrasonography provides valuable diagnostic support by assessing testicular morphology and perfusion, surgical exploration remains the definitive diagnostic and therapeutic approach.

The authors describe a term male neonate in whom a painless, firm enlargement of the right testis was detected during routine newborn assessment and subsequently confirmed to represent prenatal testicular torsion. Owing to the absence of testicular viability, right orchiectomy was performed, accompanied by prophylactic orchiopexy of the contralateral testis.

This report highlights the challenges involved in recognizing prenatal testicular torsion and underscores the importance of systematic neonatal examination, accurate differentiation from postnatal torsion, and appropriate clinical follow-up. Ongoing documentation of such cases remains crucial to advancing clinical knowledge and supporting the development of evidence-informed management strategies.

## Introduction

Neonatal testicular torsion (NTT) is an uncommon but clinically significant condition, defined by rotation of the testis and spermatic cord in the neonatal period [1,2]. This entity is broadly categorized into prenatal (intrauterine) torsion, a typically non-acute event that occurs before delivery, and postnatal torsion, which arises after delivery within the first 30 days of life [1,2]. Clinically, prenatal torsion presents as a firm, enlarged, or discolored hemiscrotum with substantial risk of ischemia, necrosis, and irreversible testicular loss, while postnatal torsion more often presents as an acute scrotum with evolving swelling, erythema, tenderness/irritability, and a time-sensitive (still limited) opportunity for salvage [1,3].

From a pathophysiologic standpoint, prenatal testicular torsion is predominantly extravaginal, as the gubernaculum remains insufficiently fixed during fetal development, allowing rotation of both the spermatic cord and the testis [1,4]. The precise etiology of extravaginal testicular torsion remains incompletely understood. It has been postulated that, in the neonatal period, inadequate fixation of the tunica vaginalis to the scrotal wall permits excessive mobility of the testis and spermatic cord, predisposing to torsion, particularly in the setting of cremasteric muscle contraction [5,6].

The reported incidence of NTT ranges from approximately 6.1 to 11.3 per 100,000 male live births, although its true incidence may be underestimated because many events occur before birth and can be clinically subtle or recognized only later as an atrophic or absent testis [1,7]. Across published series and reviews, prenatal (intrauterine) torsion accounts for the majority of NTT cases, typically representing approximately 70% to 80% of reported events [1,8]. Most cases are thought to occur in the third trimester; nevertheless, antenatal diagnosis remains uncommon because clinical and sonographic findings may be subtle or absent [4-6,9]. Accurate distinction between prenatal and postnatal torsion is essential, as they differ in pathophysiology, urgency of intervention, and recommended management strategies [3-6].

Postnatal recognition relies on careful physical examination supported by ultrasonography with Doppler [1,3]. In prenatal torsion, typical sonographic findings include reduced testicular size, irregular margins, heterogeneous or hyperechogenic echotexture, and absent Doppler perfusion; calcifications may also be present in more advanced cases [4]. However, normal or equivocal Doppler findings do not reliably exclude torsion in neonates because of low baseline testicular perfusion and operator dependence [10]. Surgical management generally involves exploration with orchiectomy of the affected testis when non-viable and consideration of contralateral orchiopexy to reduce the risk of subsequent torsion [1-3, 6].

The prognosis of prenatal testicular torsion is exceedingly poor, as testicular salvage rates are virtually negligible even when surgical exploration is performed immediately after birth [2,4,6]. Nevertheless, because the condition is unilateral in the vast majority of cases, overall endocrine function is typically preserved, with the contralateral testis providing adequate hormonal activity [2,3,6]. We present the case of a term male newborn with unilateral prenatal testicular torsion identified during routine examination at 24 hours of life. This case emphasizes careful neonatal examination and timely diagnostic assessment in differentiating prenatal from postnatal testicular torsion and guiding appropriate management.

## Case presentation

A male neonate was born to a healthy 28-year-old primigravida mother (G1P1) at 39+six weeks’ gestation by uncomplicated spontaneous vaginal delivery in a hospital setting. The mother experienced an uneventful pregnancy, with standard outcomes from prenatal laboratory tests and ultrasound examinations. There was no significant family or perinatal history. The appearance, pulse, grimace, activity, and respiration (APGAR) scores were 9, 10, and 10 at one, five, and 10 minutes, respectively. Anthropometric measurements were appropriate for gestational age, including a birth weight of 3512 g (50th percentile), a length of 47 cm (4th percentile), and a head circumference of 34.5 cm (27th percentile), according to the Fenton 2025 growth charts [11].

At the initial clinical assessment performed at 24 hours of life, marked testicular asymmetry was observed. The right hemiscrotum appeared mildly darkened, and palpation revealed a firm, non-tender testicular mass, with no associated erythema, edema, or other overt inflammatory signs (Figure 1). The right testis was enlarged and indurated, measuring approximately 2 cm in maximal diameter, and the ipsilateral cremasteric reflex was absent. The infant exhibited no signs of discomfort on examination. The contralateral testis was normally positioned, soft, and of appropriate size for age, with a preserved cremasteric reflex. No additional abnormalities were identified on the remainder of the physical examination, and the newborn remained calm, well-appearing, and hemodynamically stable throughout the evaluation.

**Figure 1:**
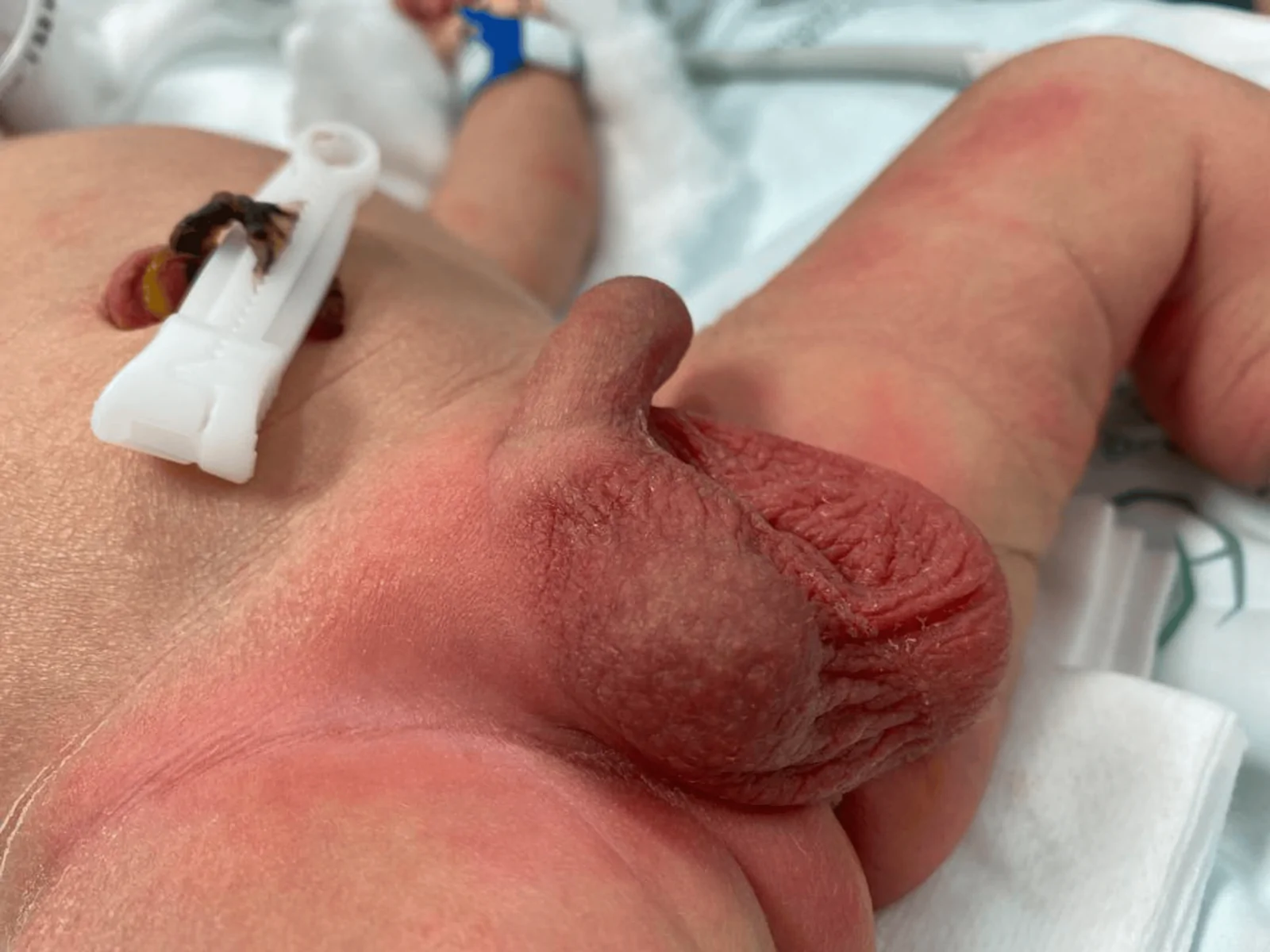
Clinical appearance of the right testis Clinical examination revealed an enlarged, firm, and darkened right testis compared with the contralateral testis.

Scrotal ultrasonography demonstrated both testes in an intrascrotal position, with an estimated volume of 0.25 cm³ for the left testis and 0.30 cm³ for the right. The left testis exhibited normal size, echotexture, and morphology, whereas the right testis appeared enlarged with an abnormal, vaguely reniform configuration (Figure 2). Color Doppler imaging revealed an absence of detectable vascular flow within the right testis and epididymis, findings strongly suggestive of right-sided testicular torsion (Figure 3).

**Figure 2:**
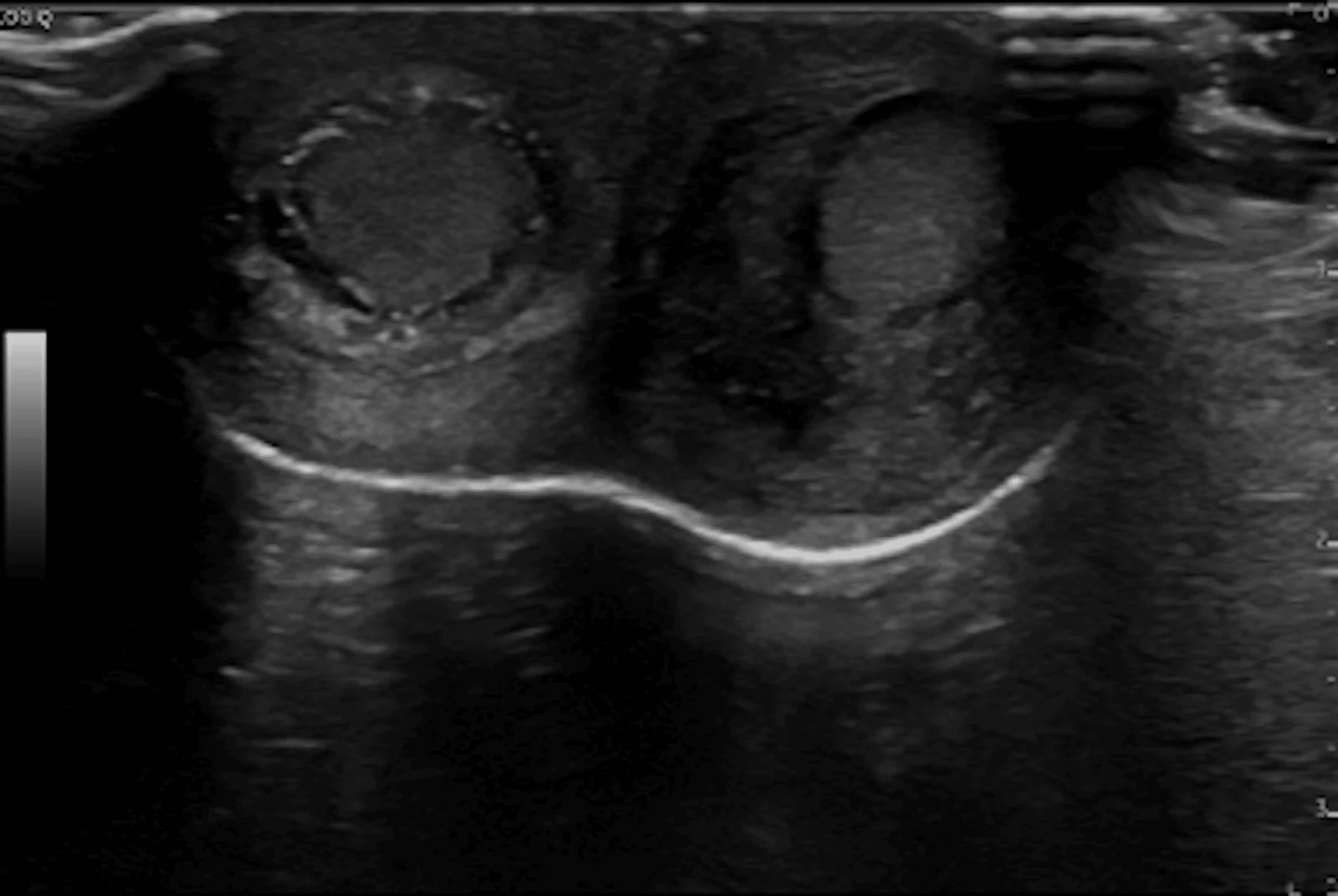
Grayscale ultrasonographic appearance of the right testis Scrotal ultrasonography demonstrated the right testis in an intrascrotal position, with abnormal, vaguely reniform morphology.

**Figure 3:**
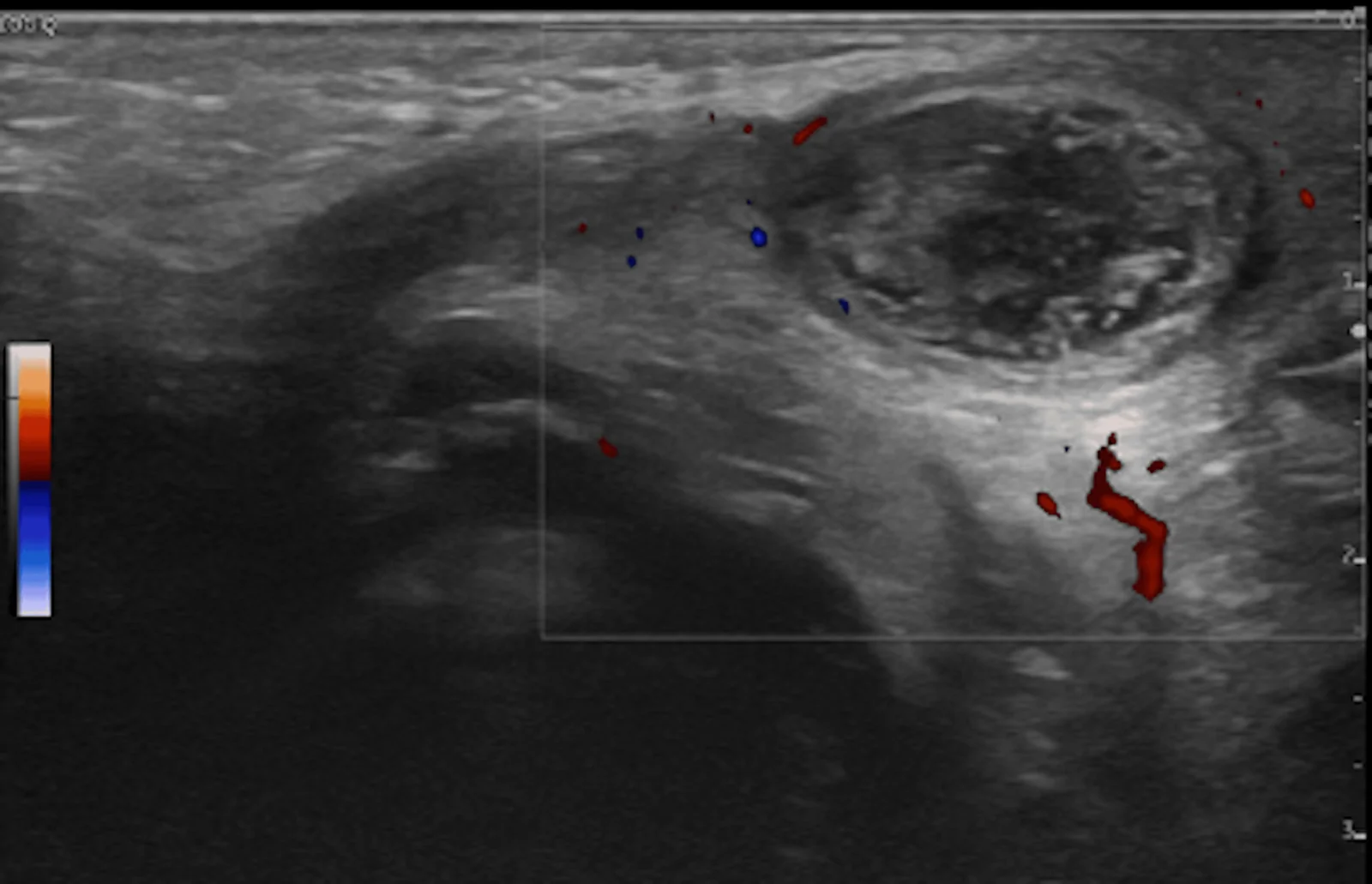
Color Doppler ultrasonography of the right testis Color Doppler ultrasonography demonstrated absence of detectable vascular flow within the right testis and epididymis, a finding consistent with right-sided testicular torsion.

Additionally, nonspecific hyperechogenic foci were identified along the inner surface of the right tunicae, an ultrasonographic feature supportive of chronic or prenatal process. No hydrocele or intrascrotal hernia was identified. On the left side, testicular echogenicity was normal, with preserved vascular flow.

The newborn was transferred to a tertiary pediatric surgical center, where surgical exploration revealed a nonviable right testis with extensive necrotic changes (Figure 4). A right orchiectomy with prophylactic fixation of the contralateral testis was performed without complications.

**Figure 4:**
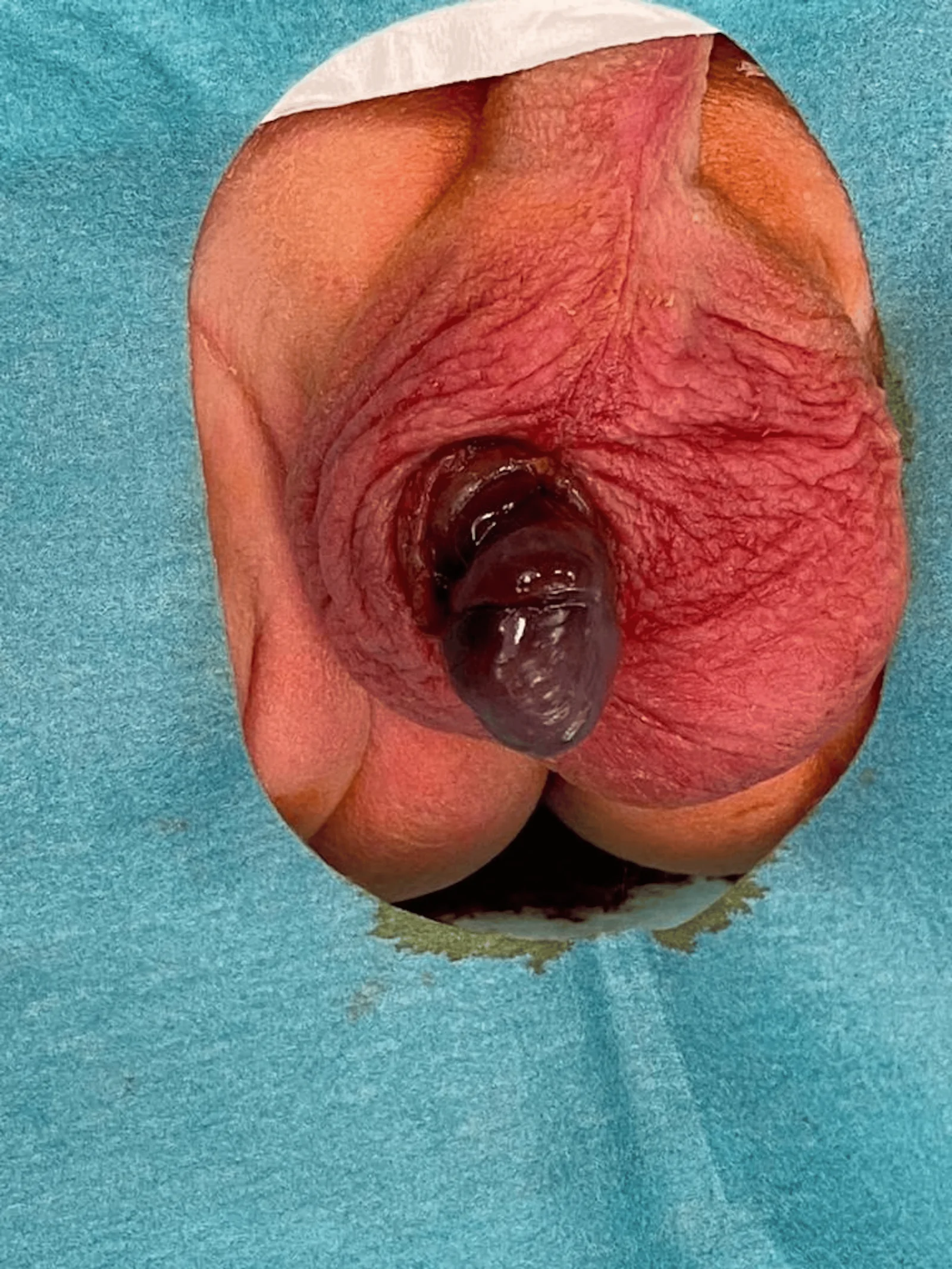
Intraoperative appearance of the right testis Intraoperative view of the right testis following surgical exploration and incision, demonstrated extensive necrotic testicular tissue.

The postoperative course was uneventful, and the infant was discharged on the second postoperative day. Histopathological examination of the excised specimen demonstrated extensive testicular necrosis, findings consistent with a non-acute process. Outpatient follow-up was scheduled, during which the option of testicular prosthesis placement was discussed with the family.

## Discussion

Neonatal testicular torsion is traditionally regarded as a rare clinical entity; however, an increasing number of reports suggest that it may be more frequently recognized in contemporary practice, likely reflecting heightened clinical awareness and improved diagnostic evaluation [1,3,7]. It is generally thought to occur either antenatally or within the first 30 days of life, although this temporal classification remains somewhat arbitrary [1,2,12]. From a clinical and prognostic standpoint, the most meaningful distinction lies between torsion occurring before birth and that arising in the early postnatal period, as prenatal torsion is associated with a substantially poorer likelihood of testicular salvage [12].

The underlying causes of prenatal testicular torsion remain poorly defined, with no specific causal factor established [2,7]. Several obstetric and perinatal circumstances, including prolonged or difficult labor, dystocia, breech presentation, fetal macrosomia, and multiple gestation, have been reported in association with prenatal torsion [1,5]. However, these associations are inconsistent across published series and should not be interpreted as evidence of causality [2]. In our patient, none of these previously reported circumstances were identified, further illustrating the absence of a consistent clinical risk profile for prenatal torsion.

Consistent with this distinction, the clinical presentation of NTT is highly variable and often does not correlate reliably with testicular viability at the time of diagnosis [1,12,13]. Physical findings may range from subtle scrotal asymmetry to more conspicuous signs, including scrotal discoloration and testicular induration, largely reflecting the duration and severity of ischemic injury [1,5-7]. While acute perinatal torsion may present with irritability or feeding intolerance, these features are often absent in prenatal torsion [1-3]. In the present case, a painless, enlarged, and firm right testis was identified in an otherwise asymptomatic newborn, highlighting the critical role of meticulous physical examination in the early neonatal period.

Scrotal ultrasonography plays a pivotal role in the evaluation of suspected perinatal testicular torsion by assessing testicular morphology and perfusion and aiding in the exclusion of alternative diagnoses such as epididymitis, hydrocele, indirect inguinal hernia, and testicular neoplasms [1,14]. Although Doppler ultrasonography provides valuable diagnostic information, its utility remains adjunctive [1,5-7,10]. Imaging findings alone are insufficient to definitively establish testicular viability or to obviate the need for surgical exploration, which remains the diagnostic and therapeutic gold standard [1,7-8,10].

The management of prenatal testicular torsion remains controversial, particularly regarding the optimal timing of surgical exploration and the role of prophylactic fixation of the contralateral, unaffected testis [2,3,6,13]. Given the extremely low probability of testicular salvage in presumed antenatal torsion, some authors advocate delayed surgical intervention, allowing optimization of anesthetic and operative conditions [2,4-6]. Conversely, others favor timely exploration because of the potential, albeit rare, risk of synchronous or asynchronous bilateral torsion [6,12]. Contralateral orchiopexy is therefore widely adopted in clinical practice to minimize the risk of subsequent torsion and anorchia [3,5-7,10]. However, the evidence supporting routine prophylactic fixation remains limited [1,10]. Recurrence following orchiopexy is uncommon, although cases have been reported several years after the initial procedure [15]. In the present case, despite timely referral and appropriate surgical management, testicular salvage was not feasible, a finding consistent with the well-documented poor viability associated with presumed prenatal torsion. Orchiectomy of the nonviable testis was therefore performed, along with prophylactic fixation of the contralateral testis.

This case is presented both for its rarity and to emphasize the critical importance of thorough physical examination in the neonatal period. Prenatal testicular torsion often results in irreversible ischemic injury, leading to testicular atrophy or degeneration, and may go unrecognized in the absence of careful clinical assessment [6]. Moreover, accurate differentiation from postnatal torsion is essential, as the latter represents a true surgical emergency in which prompt intervention may significantly improve the likelihood of testicular preservation [3,6].

Given the consistently low salvage rates associated with prenatal torsion and the absence of standardized management guidelines [5-7,12,13], the reporting of additional cases remains valuable. Ultimately, such reports reinforce the necessity for heightened clinical vigilance, early recognition, and structured long-term follow-up in affected patients.

## Conclusions

This case highlights the diagnostic challenges inherent to prenatal testicular torsion and underscores the critical importance of meticulous physical examination during the neonatal period. Prenatal torsion frequently results in irreversible ischemic injury, often rendering the affected testis nonviable by the time of diagnosis and allowing the condition to remain clinically silent unless actively sought. Accurate distinction between prenatal and postnatal torsion is therefore essential, as postnatal torsion constitutes a true surgical emergency in which timely intervention may substantially improve the likelihood of testicular preservation.

Given the rarity of perinatal testicular torsion, the consistently low rates of testicular salvage in prenatal cases, and the absence of standardized management guidelines, the reporting of additional cases remains of considerable value. Limitations imposed by ethical considerations, heterogeneous clinical presentations, and low disease prevalence preclude randomized controlled trials, resulting in management strategies that are largely consensus-based. Continued documentation of NTT may contribute to a more robust understanding of this uncommon entity and support the development of multicenter, evidence-informed recommendations.
